# Meta-regression models to address heterogeneity and inconsistency in network meta-analysis of survival outcomes

**DOI:** 10.1186/1471-2288-12-152

**Published:** 2012-10-08

**Authors:** Jeroen P Jansen, Shannon Cope

**Affiliations:** 1 MAPI Consultancy, 180 Canal Street, Suite 503, Boston, MA, 02114, USA; 2 Tufts University School of Medicine, Boston, MA, USA

## Abstract

**Background:**

Recently, network meta-analysis of survival data with a multidimensional treatment effect was introduced. With these models the hazard ratio is not assumed to be constant over time, thereby reducing the possibility of violating transitivity in indirect comparisons. However, bias is still present if there are systematic differences in treatment effect modifiers across comparisons.

**Methods:**

In this paper we present multidimensional network meta-analysis models for time-to-event data that are extended with covariates to explain heterogeneity and adjust for confounding bias in the synthesis of evidence networks of randomized controlled trials. The impact of a covariate on the treatment effect can be assumed to be treatment specific or constant for all treatments compared.

**Results:**

An illustrative example analysis is presented for a network of randomized controlled trials evaluating different interventions for advanced melanoma. Incorporating a covariate related to the study date resulted in different estimates for the hazard ratios over time than an analysis without this covariate, indicating the importance of adjusting for changes in contextual factors over time.

**Conclusion:**

Adding treatment-by-covariate interactions to multidimensional network meta-analysis models for published survival curves can be worthwhile to explain systematic differences across comparisons, thereby reducing inconsistencies and bias. An additional advantage is that heterogeneity in treatment effects can be explored.

## Background

Healthcare decision-making requires comparisons of relevant competing treatments for a particular disease state. In the absence of trials involving a direct comparison of interventions, an indirect comparison can provide useful evidence for the treatment effects between competing interventions [1-6]. Even when direct evidence is available, combining consistent direct and indirect estimates will result in more precise treatment effect estimates [2-6]. In general, if the evidence base consists of multiple randomized controlled trials (RCTs) with each trial having at least one intervention in common with another, this network of RCTs can be synthesized with meta-analysis techniques: a network meta-analysis (or mixed treatment comparison meta-analysis) [1-6].

Traditional (network) meta-analyses for survival outcomes are based on hazard ratios (HR) and rely on the proportional hazards assumption, which is implausible if the hazard functions of competing interventions cross. Ouwens et al. (2011) and Jansen (2011) presented methods for (network) meta-analysis of survival data using a multidimensional treatment effect as an alternative to the synthesis of the constant HRs [7,8]. The hazard functions of the interventions in a trial are modeled using known parametric survival functions (e.g. Weibull or Gompertz) or fractional polynomials and the difference in the parameters are considered the multidimensional treatment effect, which are synthesized (and indirectly compared) across studies. In essence, with this approach the treatment effects are represented by multiple parameters rather than a single parameter.

For network meta-analysis it is important that transitivity (i.e. the consistency assumption) holds for the treatment effect measure of interest [2,3,9]. Violations of the proportional hazards assumption will compromise transitivity and can result in biased indirect and mixed treatment comparisons of survival outcomes [8]. By incorporating additional parameters for the treatment effect as proposed with the methods by Ouwens et al. and Jansen, the proportional hazards assumption is relaxed and indirect comparisons are less likely to be biased [7,8]. However, violation of the proportional hazards assumption is not the only possible source of bias. Since randomization of patients does not hold across trials, there might be an imbalance in patient or study level covariates across comparisons [4-6,9]. If these covariates are effect-modifiers of the (time varying) HRs, transitivity will be violated and the network meta-analysis will result in biased estimates, despite modeling a multidimensional treatment effect.

In this paper, the models proposed by Ouwens et al. and Jansen are extended with treatment-by-covariate interactions to adjust for confounding bias due to systematic differences in treatment effect modifiers across comparisons. These models also have the advantage of explaining heterogeneity in the treatment effects. The method is illustrated with an example.

## Methods

### Multidimensional network meta-analysis models for survival data

If AB trials and AC trials are comparable in terms of effect modifiers (i.e. covariates that affect the treatment effect) then an indirect estimate for the treatment effect of C versus B (*d*_*BC*_) can be obtained from the estimates of the effect of B versus A (*d*_*AB*_) and the effect of C versus A (*d*_*AC*_): *d*_*BC*_ *= d*_*AC*_*- d*_*AB*_; as such, transitivity holds [2,3,6]. For an arm-based model with the outcome of treatment *x* as a function of time, *f*_*x*_*(t)* where x = *A, B, or C* and *t* represents time, this consistency assumption translates into:

(1) $$\left(f_{C}\left(t\right)-f_{B}\left(t\right))=\left(f_{C}\left(t\right)-f_{A}\left(t\right)\right)-(f_{B}\left(t\right)-f_{A}\left(t\right)\right)$$

The hazard function is of central interest to summarize survival or time-to-event data. The hazard function describes the instantaneous event (e.g. death) rate at any point in time. A random effects network meta-analysis model for hazard rates by treatment arm without assuming a particular distribution for the hazard function over time can defined according to:

(2) $$\begin{array}{l} \ln(h_{\mathrm{jkt}})=\left\{ \begin{array}{c} \mu_{\mathrm{jbt}}\;\\ \mu_{\mathrm{jbt}}+\delta_{\mathrm{jbk}} \end{array}\right.\;\begin{array}{c} b=\text{A,B,C, if}\;k=b\\ \text{if}\;k\;\text{'alphabetically after'}\;b \end{array}\\ \;\delta_{\mathrm{jbk}}\sim normal(d_{\mathrm{bk}},\sigma^{2})=normal(d_{\mathrm{Ak}}-d_{\mathrm{Ab}},\sigma^{2}) \end{array}$$

where *h*_*jkt*_ reflect the underlying hazard rate in trial *j* for intervention *k* at time point *t*. *μ*_*jbt*_ are the study *j* specific hazard rates at time *t* with comparator treatment *b*. *δ*_*jbk*_ reflects the study specific constant log HRs of treatment *k* relative to comparator treatment *b* and are drawn from a normal distribution with the pooled estimates expressed in terms of the overall reference treatment *A: d*_*bk*_=*d*_*Ak*_ − *d*_*Ab*_ with *d*_*AA*_ = 0. Variance *σ*^2^ reflects the heterogeneity across studies. As an alternative to a non-parametric baseline hazard function, the development of the hazard rate over time can be described by parametric functions, such as Weibull or Gompertz.

#### Two-dimensional treatment effects without covariates (Model 1)

Ouwens et al. and Jansen introduced network meta-analysis models for parametric hazard functions [7,8]. A network meta-analysis model for the hazard rate with a two dimensional treatment effect can be defined according to:

(3) $$\begin{array}{l} \ln(h_{\mathrm{jkt}})=\beta_{0jk}+\beta_{1jk}t^{p}\;\text{with}\;t^{0}=\text{ln(t)}\;\\ \left(\begin{array}{c} \beta_{0jk}\\ \beta_{1jk} \end{array}\right)=\left\{ \begin{array}{c} \left(\begin{array}{c} \mu_{0jb}\\ \mu_{1jb} \end{array}\right)\;\\ \left(\begin{array}{c} \mu_{0jb}\\ \mu_{1jb} \end{array}\right)+\left(\begin{array}{c} \delta_{0jbk}\\ d_{1Ak}-d_{1Ab} \end{array}\right)\; \end{array}\begin{array}{c} b=\text{A,B,C, if}\;k=b\\ \text{if}\;k\;\text{'alphabetically after'}\;b \end{array}\right.\\ \;\delta_{0jbk}\sim normal(d_{0Ak}-d_{0Ab},\sigma^{2}) \end{array}$$

where *h*_*jkt*_ again reflects the underlying hazard rate in trial *j* for intervention *k* at time point *t* and is now described as a function of time *t* with *p*=*{*-2,-1,-0.5,0,0.5,1,2,3*}* and *t*^0^=ln(t) with treatment and study specific scale and shape parameters *β*_0*jk*_ and *β*_1*jk*_. (In the example presented below details on the likelihood and data structure are provided). If *β*_1*jk*_ equals 0, a constant log hazard function is obtained, reflecting exponentially distributed survival times. If *β*_1*jk*_ ≠ 0 and *p* = 1 a linear log hazard function is obtained which corresponds to a Gompertz survival function [8]. If *β*_1*jk*_ ≠ 0 and *p* = 0 a Weibull hazard function is obtained. The vectors $\left(\begin{array}{c} \mu_{0jb}\\ \mu_{1jb} \end{array}\right)$ are trial specific and reflect the true underlying scale and shape parameters of the comparator treatment *b*. *δ*_0*jbk*_ is the study specific difference in the scale parameter *β*_0_ of the log hazard curve for treatment *k* relative to comparator treatment *b. δ*_0*jbk*_ are drawn from a normal distribution with the pooled estimates expressed in terms of the overall reference treatment *A*: *d*_0*Ak*_ − *d*_0*Ab*_ with *d*_0*AA*_ = 0. The parameters *d*_0*Ak*_ correspond to the treatment effect of k relative to overall reference treatment *A* with a proportional hazard model. The pooled difference in the shape parameter *β*_1_ of the log hazard curve for treatment *k* relative to comparator treatment *b* is expressed as *d*_1*Ak*_ − *d*_1*Ab*_ with *d*_1*AA*_ = 0. *d*_1*Ak*_ reflects the change in the log HR over time. For a proportional hazards model *d*_1*Ak*_ equals 0. By incorporating *d*_1*Ak*_ in addition to *d*_0*Ak*_ a multidimensional treatment effect is used. For additional flexibility, the first order fractional polynomial model can be generalized to a 2^nd^ order fractional polynomial model, representing 3-dimensional treatment effects [8].

Variance *σ*_*0*_^2^ reflects the heterogeneity in the difference in the scale parameters across studies. A random effects model with only a heterogeneity parameter for *d*_0*Ak*_ implies that the between study variance of the log hazard ratios remains constant over time.

#### Two-dimensional treatment effects with treatment specific covariate interactions (Model 2)

Since randomization only holds within a trial, the distribution of covariates may vary across studies for a particular type of comparison, as well as between different types of comparisons. Variation in treatment effect modifiers across studies within comparisons results in heterogeneity and an imbalance in effect modifiers between different types of comparisons results in violations of the consistency assumption. [2-4,6,9,10]. Network meta-analysis models can be extended with treatment-by-covariate interactions in an attempt to adjust for this confounding bias or to explore sources of heterogeneity [9-14].

In line with previous meta-regression models for network meta-analysis where the treatment effect acts on one parameter [10], model 1 can be extended with study level covariates to explore treatment-by-covariate interactions. The specification assumes that all treatment-by-covariate interactions are different for each treatment *k* relative to overall reference treatment *A*:

(4) $$\begin{array}{l} \ln(h_{\mathrm{jkt}})=\beta_{0jk}+\beta_{1jk}t^{p}\;\text{with}\;t^{0}=\text{ln(t)}\;\\ \left(\begin{array}{c} \beta_{0jk}\\ \beta_{1jk} \end{array}\right)=\left\{ \begin{array}{c} \left(\begin{array}{c} \mu_{0jb}\\ \mu_{1jb} \end{array}\right)\;\\ \left(\begin{array}{c} \mu_{0jb}\\ \mu_{1jb} \end{array}\right)+\left(\begin{array}{c} \delta_{0jbk}\\ d_{1Ak}-d_{1Ab} \end{array}\right)\; \end{array}\begin{array}{c} b=\text{A,B,C, if}\;k=b\\ \text{if}\;k\;\text{'alphabetically after'}\;b \end{array}\right.\\ \;\delta_{0jbk}\sim normal(d_{0bk}+\beta_{\mathrm{xbk}}X_{j},{\sigma_{0}}^{2})\\ \;=normal(d_{0Ak}-d_{0Ab}+(\beta_{\mathrm{xAk}}-\beta_{\mathrm{xAb}})X_{j},{\sigma_{0}}^{2}) \end{array}$$

*β*_*xbk*_ reflects the impact of study level covariate *X*_*j*_ on the difference in the scale parameters of the hazard functions with treatment *k* relative to control treatment *b*. Now *d*_0*bk*_ is the difference in scale treatment k relative to *b* when the covariate value equals zero. *Since β*_*xbk*_ = *β*_*xAk*_ − *β*_*xAb*_ with *β*_*xAA*_ = 0, *k − 1* different and independent regression coefficients for *β*_*xAk*_ will be estimated with the model. As an alternative to independent treatment-by-covariate interactions, one can also assume exchangeable interaction effects [10].

#### Two-dimensional treatment effects with constant covariate interaction (Model 3)

One can also assume that the impact of the covariate *X*_*j*_ on the scale parameter of each treatment *k* relative to *A* is the same for all treatments. This assumption can be defended when treatments indirectly compared are all from the same class and there is no (biological) reason to assume that a patient characteristic, or any other contextual aspect of the study, modifies treatment effects differently for the different drugs compared. Furthermore, the assumption of constant treatment-by-covariate interaction can also be useful when evaluating the impact of study (design) characteristics (or bias) on treatment effects. [10,14]. The corresponding network meta-analysis model will be:

(5) $$\begin{array}{l} \ln(h_{\mathrm{jkt}})=\beta_{0jk}+\beta_{1jk}t^{p}\;\text{with}\;t^{0}=\text{ln(t)}\;\\ \left(\begin{array}{c} \beta_{0jk}\\ \beta_{1jk} \end{array}\right)=\left\{ \begin{array}{c} \left(\begin{array}{c} \mu_{0jb}\\ \mu_{1jb} \end{array}\right)\;\\ \left(\begin{array}{c} \mu_{0jb}\\ \mu_{1jb} \end{array}\right)+\left(\begin{array}{c} \delta_{0jbk}\\ d_{1Ak}-d_{1Ab} \end{array}\right)\; \end{array}\begin{array}{c} b=\text{A,B,C, if}\;k=b\\ \text{if}\;k\;\text{'alphabetically after'}\;b \end{array}\right.\\ \delta_{0jbk}\sim \left\{ \begin{array}{c} normal\left(d_{0Ak}-d_{0Ab}+\beta_{x}X_{j},{\sigma_{0}}^{2}\right)\;\text{if}\;b=\text{A}\\ normal\left(d_{0Ak}-d_{0Ab},{\sigma_{0}}^{2}\right)\;\text{if}\;b\neq \text{A} \end{array}\right. \end{array}$$

Since for each treatment *k* relative to *A* the impact of the covariate is the same, *β*_*x*_*X*_*j*_ will cancel out for the comparison of treatment *k* relative to *b* when *b ≠* A

(6) $$normal\left(\left(d_{0Ak}+\beta_{x}X_{j}\right)-\left(d_{0Ab}+\beta_{x}X_{j}\right),{\sigma_{0}}^{2}\right)=normal\left(d_{0bk},{\sigma_{0}}^{2}\right)$$

**Figure 1:**
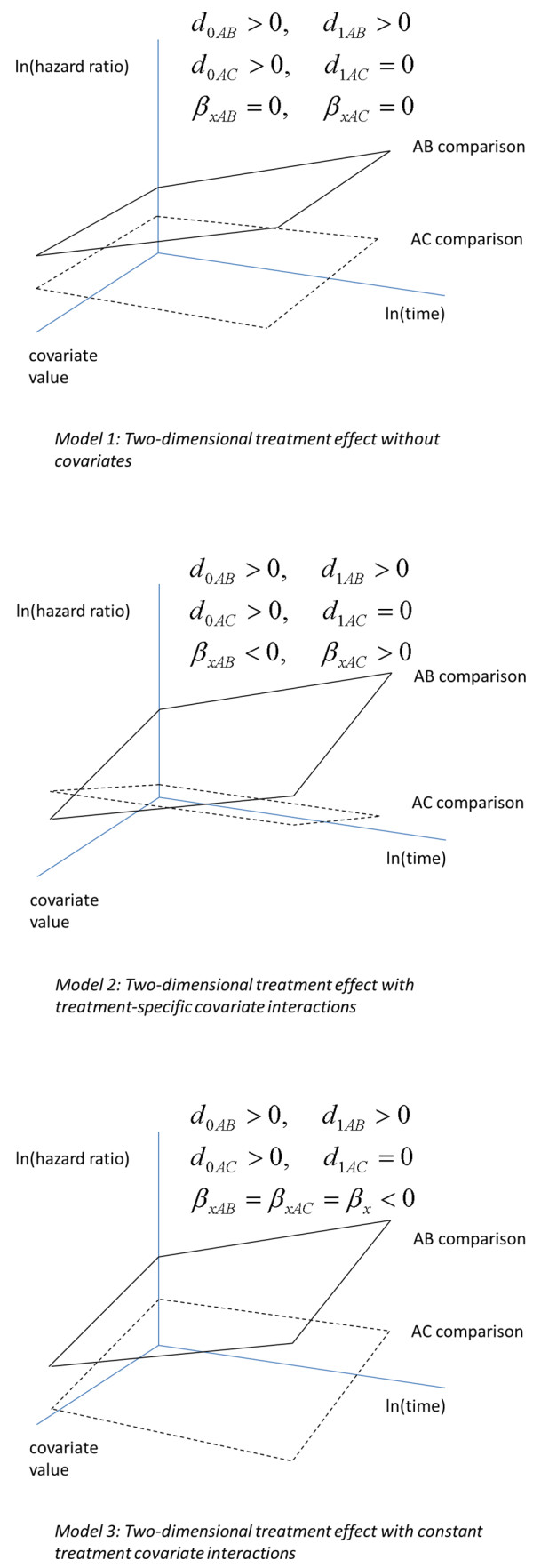
**Graphical representation of two-dimensional network meta-analysis model for survival data without treatment-by-covariate interaction (A); two-dimensional network meta-analysis model with treatment specific covariate interactions (B); two-dimensional network meta-analysis model with constant covariate interaction (C).** (See Model 1–3 and text for explanation)

Figure 1 illustrates the results of a network meta-analysis of *AB* and *AC* studies according to Models 1–3 assuming the hazard over time follows a Weibull distribution, i.e. *p = 0*. The *AB* and *AC* planes reflect the pooled effect estimates of treatment *B* and *C* relative to *A* as a function of time and a covariate value. The vertical axis represents model parameters *d*_0*Ak*_ with *k = B, C,* corresponding to the log-HR of treatment *B* and *C* relative to *A* at time *t* = 0 and covariate value *X = 0*. The slope of the *AB* and *AC* planes as a function of ln(time) represents parameter *d*_1*Ak*_, the impact of time on the log HR of treatment *k* relative to *A*. The slope of the *AB* and *AC* planes as a function of the covariate value represents *β*_*xAk*_, which is the impact of covariate *X* on the treatment effect parameter *d*_0*Ak*_ (the scale). Figure 1A represents Model 1 where it is assumed that the covariate is not an effect modifier of *d*_0*Ak*_ and therefore *β*_*xAk*_ = 0. In Figure 1B the effect of the covariate for the *AB* comparison is different from the *AC* comparison (Model 2). Figure 1C reflects Model 3 with the same effect of the covariate for the *AB* and the *AC* comparison.

The random effects Models 1–3 treat multiple-arm trials (>2 treatments) without taking account of the correlations between the trial specific *δ*s that they estimate. Bayesian random effects models with a heterogeneity parameter for *d*_0*Ak*_ can be easily extended to fit trials with 3 or more treatment arms by decomposing a multivariate normal distribution as a series of conditional univariate distributions [9,13]:

(7) δ0jbk1⋮δ0jbkp~normald0bk1⋮d0bkp,σ2σ22⋯σ22σ22σ2⋯σ22⋮⋮⋱⋮σ22σ22⋯σ2

then the conditional univariate distributions for arm *i* given the previous *1,….(i-1)* arms are:

(8) $${\delta_{0jbk}}_{i}|\left(\begin{array}{c} {\delta_{0jbk}}_{1}\\ \vdots \\ {\delta_{0jbk}}_{i-1} \end{array}\right)\sim normal\left(d_{0bk_{i}}+\frac{1}{i}\sum_{j=1}^{i-1}\left({\delta_{0jbk}}_{j}-d_{0bk_{j}}\right),\frac{\left(i+1\right)}{2i}\sigma^{2}\right)$$

#### Higher dimensional models with heterogeneity and covariate effects acting on multiple treatment effect parameters

Models 2 and 3 have treatment effects on a scale and one shape parameter, and make the simplifying assumption that the heterogeneity and covariate only interact with the treatment effects in terms of scale. Two-dimensional random effects models with additional heterogeneity parameters for treatment in terms of shape have the flexibility to capture between study variance regarding changes in the log HRs over time [8]. Building upon the higher-dimensional network meta-analysis models proposed by Jansen [8], a general formulation is a model with heterogeneity and covariate effects that act on 3 treatment effect parameters (i.e. 1 scale and 2 shape parameters) as presented at the end of this page.

(9) $$\begin{array}{l} \ln(h_{\mathrm{jkt}})=\left\{ \begin{array}{c} \beta_{0jk}+\beta_{1jk}t^{p_{1}}+\beta_{2jk}t^{p_{2}}\;\\ \beta_{0jk}+\beta_{1jk}t^{p_{1}}+\beta_{2jk}t^{p_{1}}\ln(t) \end{array}\right.\;\begin{array}{c} p_{1}\neq p_{2}\\ p_{1}=p_{2} \end{array}\;\text{with}\;t^{0}=\ln(t)\\ \left(\begin{array}{c} \beta_{0jk}\\ \beta_{1jk}\\ \beta_{2jk} \end{array}\right)=\left\{ \begin{array}{c} \left(\begin{array}{c} \mu_{0jb}\\ \mu_{1jb}\\ \mu_{2jb} \end{array}\right)\;\;b=A,B,C,\ldots ,\;\text{if}k=b\\ \left(\begin{array}{c} \mu_{0jb}\\ \mu_{1jb}\\ \mu_{2jb} \end{array}\right)+\left(\begin{array}{c} \delta_{0jbk}\\ \delta_{1jbk}\\ \delta_{2jbk} \end{array}\right)\;\text{if}\;k\;\text{'alphabetically' after}\;b. \end{array}\right.\\ \left(\begin{array}{c} \delta_{0jbk}\\ \delta_{1jbk}\\ \delta_{2jbk} \end{array}\right)\sim normal\left(\left(\begin{array}{c} d_{0Ak}\\ d_{1Ak}\\ d_{2Ak} \end{array}\right)-\left(\begin{array}{c} d_{0Ab}\\ d_{1Ab}\\ d_{2Ab} \end{array}\right)+\left(\begin{array}{c} \beta_{\mathrm{xAk}}^{0}\\ \beta_{\mathrm{xAk}}^{}\\ \beta_{\mathrm{xAk}}^{2} \end{array}\right)-\left(\begin{array}{c} \beta_{\mathrm{xAb}}^{0}\\ \beta_{\mathrm{xAb}}^{1}\\ \beta_{\mathrm{xAb}}^{2} \end{array}\right),\Sigma \right)\;\Sigma =\left(\begin{array}{ccc} \sigma_{0}^{2} & \sigma_{0}\sigma_{1}\rho_{01} & \sigma_{0}\sigma_{2}\rho_{02}\\ \sigma_{0}\sigma_{1}\rho_{01} & \sigma_{1}^{2} & \sigma_{1}\sigma_{2}\rho_{12}\\ \sigma_{0}\sigma_{2}\rho_{02} & \sigma_{1}\sigma_{2}\rho_{12} & \sigma_{2}^{2} \end{array}\right)\; \end{array}$$

$\left(\begin{array}{c} \beta_{\mathrm{xAk}}^{0}\\ \beta_{\mathrm{xAk}}^{1}\\ \beta_{\mathrm{xAk}}^{2} \end{array}\right)$ reflect the impact of study level covariate *X*_*j*_ on the pooled treatment effects in terms of scale, *δ*_0*jbk*_, and shape *δ*_1*jbk*_ and *δ*_2*jbk*_, with treatment *k* relative to control treatment *b*.

Σ is the between study covariance matrix with *σ*_0_, *σ*_1_, *σ*_2_ representing the heterogeneity in treatments effects *δ*_0*jbk*_, *δ*_1*jbk*_ and *δ*_2*jbk*_ respectively. *ρ*_01_, *ρ*_02_ and *ρ*_12_ are the correlations between these parameters. Although such a general model is very flexible to explore heterogeneity and inconsistency, identifiability is expected to be a challenge.

### Illustrative example

An example of the models is presented for a network meta-analysis of treatment of advanced (stage IIIc or IV) melanoma. Although the interventions compared in the network in this example do not reflect the latest treatment options for melanoma, it provides a useful illustration of the survival models with covariates.

**Figure 2:**
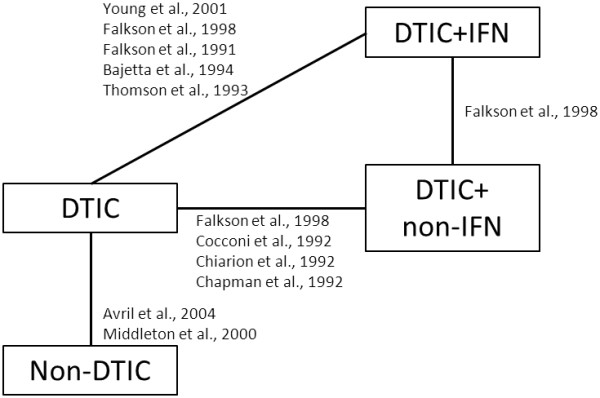
Network of randomized controlled trials

In the early stages of melanoma, surgery presents a potential curative option. However, for unresectable late stages, the mainstay of treatment is (experimental) systemic therapy. A literature search identified 10 RCTs evaluating the following 4 treatment groups: dacarbazine (DTIC) monotherapy, DTIC + Interferon (IFN), DTIC + Non-IFN, and Non-DTIC [15-24]. The network of RCTs is presented in Figure 2.

**Figure 3:**
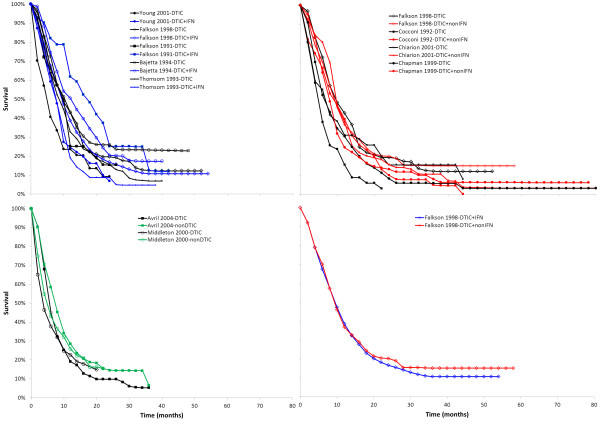
Survival as observed in individual studies with different treatment comparisons

For each treatment arm in each study the reported Kaplan-Meier curves were digitized (DigitizeIt; http://www.digitizeit.de/) and the data extracted from these trials were included in the network meta-analysis. In Figure 3 the scanned survival proportions are presented according to each direct comparison available. This aggregate data was analyzed using two-dimensional network meta-analysis models. Whilst network meta-analysis can be performed with a frequentist or a Bayesian approach, for this manuscript we focus on the Bayesian approach. Within the Bayesian framework, analyses consist of data, likelihood, parameters, and a model.

The survival curves in Figure 3 can be divided into *q* consecutive intervals over the follow-up period: [*t*_1_, *t*_2_], (*t*_2_, *t*_3_], …, (*t*_*q*_, *t*_*q*+1_] with *t*_1_=0. For each time interval *m* =1,2,3,…,*.q* extracted survival proportions were used to calculate the patients at risk at the beginning of that interval and incident number of deaths. (A more specific explanation is provided in the Additional file 1 of this paper.) A binomial likelihood distribution of the incident events for every interval can be described according to:

(10) $$r_{\mathrm{jkt}}\sim bin\left(p_{\mathrm{jkt}},n_{\mathrm{jkt}}\right)$$

Where *r*_*jkt*_ is the observed number of events in the m^th^ interval ending at time point *t*_*m*+1_ for treatment *k* in study *j*. *n*_*jkt*_ is the number of subjects at risk just before the start of that interval adjusted for the subjects censored in the interval. *p*_*jkt*_ is the corresponding underlying event probability. When the time intervals are relatively short, the hazard rate *h*_*jkt*_ at time point *t* for treatment *k* in study *j* can be assumed to be constant for any time point within the corresponding m^th^ time interval. The hazard rate corresponding to *p*_*jkt*_ for the m^th^ interval can be standardized by the unit of time used for the analysis (e.g. months) according to: *h*_*jkt*_ = − ln(1 − *p*_*jkt*_)/*Δt*_*jkt*_ where Δ*t*_*jkt*_ is the length of the interval. For the model estimation we assign this underlying hazard to time point *t*_*m*+1_.

Study date can be considered a proxy for potential changes over time in (background) medical care and other contextual factors that influence the treatment effects. For the included RCTs, there is variation within and between different types of comparisons regarding study date. The impact of study date on treatment effects was evaluated with the following meta-regression models: 1) random and fixed effects Weibull (*p = 0*) survival models without treatment-by-covariate interaction (Model 1; Figure 1A); 2) random and fixed effects Weibull survival models with treatment specific covariate interaction terms (Model 2; Figure 1B); 3) random and fixed effects Weibull survival models with a constant treatment-by-covariate interaction (Model 3; Figure 1C). Only Weibull models were used because the ln (−ln(Survival)) versus ln(time) showed linear relations for the different studies, indicating Weibull models are appropriate [25].

Here below the prior distributions are presented as used for the meta-analysis.

(11) $$\begin{array}{l} \left(\begin{array}{c} \mu_{0jb}\\ \mu_{1jb} \end{array}\right)\sim normal\left(\left(\begin{array}{c} 0\\ 0 \end{array}\right),T_{\mu }\right)\;T_{\mu }=\left(\begin{array}{cc} 10^{4} & 0\\ 0 & 10^{4} \end{array}\right)\\ \left(\begin{array}{c} d_{0Ak}\\ d_{1Ak} \end{array}\right)\sim normal\left(\left(\begin{array}{c} 0\\ 0 \end{array}\right),T_{d}\right)\;T_{d}=\left(\begin{array}{cc} 10^{4} & 0\\ 0 & 10^{4} \end{array}\right)\\ \beta_{\mathrm{xAk}}\sim normal(0,10^{4})\\ \sigma_{0}\sim uniform(0,2)\; \end{array}$$

With constant covariate interaction, *β*_*xAk*_ ∼ *normal*(0, 10^4^) will be replaced with *β*_*x*_ ∼ *normal*(0, 10^4^). With the fixed effects model, it is not necessary to define a prior distribution for *σ*_0_.

The parameters of the different models were estimated using a Markov Chain Monte Carlo (MCMC) method as implemented in the WinBUGS software package [26]. (See Additional file 1 for the code) The first 80,000 iterations from the WinBUGS sampler were discarded as ‘burn-in’ and the inferences were based on an additional 50,000 iterations using two chains. Convergence of the chains was confirmed by the Gelman-Rubin statistic.

The deviance information criterion (DIC) was used to compare the goodness-of-fit of the different models [27,28]. DIC provides a measure of model fit that penalizes model complexity according to $DIC=\overset{Â¯}{D}+pD,pD=\overset{Â¯}{D}-\hat{D}$[28]. $\overset{Â¯}{D}$ is the posterior mean residual deviance [28], *pD* is the ‘effective number of parameters’ and $\hat{D}$ is the deviance evaluated at the posterior mean of the model parameters. In general, a more complex model will result in a better fit to the data, demonstrating a smaller residual deviance. The model with the lowest DIC is the model providing the ‘best’ fit to the data adjusted for the number of parameters.

## Results

### Illustrative example

In Table 1 the model fit statistics for the different models are presented. The random effects model without covariates was associated with a smaller residual deviance than the fixed effects model without covariates. Taking into account the increased model complexity of the random effects approach, the DIC with the random effects model is also lower. We prefer the random effects model over the fixed effects model. (We take the position that in principle a random effects model is preferred over a fixed effects model because there is often cause to suspect heterogeneity. Hence, in a situation when the DIC with the more complex random effects model is comparable to the fixed effects model, suggesting there is no strong evidence against the fixed effects model on statistical grounds, we might still prefer to use the random effects model to obtain a conservative estimate. In a situation when there is not sufficient data to estimate a heterogeneity parameter, a fixed effects model is preferred.) Adding treatment specific covariate interaction terms to the random effects model improved the residual deviance, but resulted in a similar DIC (1492.5) due to the greater number of model parameters. Treatment constant interaction effects for the random effects model resulted in a similar residual deviance, but the DIC was arguably somewhat more favorable (1490.7) because of the less complex model formulation. For the fixed effects model, extension with treatment specific covariate interaction terms improved both the residual deviance and the DIC, suggesting that factors associated with study date (partly) explain the heterogeneity in the scale related treatment effects. A fixed effect model with a constant covariate interaction term showed a comparable model fit.

**Table 1:** Goodness of fit estimates for fixed effects and random effects network meta-analysis models

**Model**	**Dbar**	**Dhat**	**pD**	**DIC**
Random effects Weibull model without covariates	1462.4	1432.3	30.1	1492.5
Fixed effects Weibull model without covariates	1468.9	1443.0	25.9	1494.8
Models with study data as covariate				
Random effects Weibull model with treatment specific covariate interactions	1460.5	1428.5	32.0	1492.5
Fixed effects Weibull model with treatment specific covariate interactions	1464.7	1436.7	28.0	1492.7
Random effects Weibull model with constant treatment covariate interactions	1459.7	1428.7	31.0	1490.7
Fixed effects Weibull model with constant treatment covariate interactions	1466.7	1439.9	26.8	1493.5

Dbar = posterior mean residual deviance.

Dhat = deviance evaluated at the posterior mean of the model parameters.

pD = the effective number of parameters as a measure of model complexity.

DIC = Deviance Information Criteria.

Table 2 provides parameter estimates for the fixed and random effects model without covariates (Model 1), as well as parameter estimates for the random effects model with treatment specific covariate interaction terms (Model 2) and the random effects model with a constant covariate interaction term (Model 3). Although credible intervals for the treatment effects in terms of shape include the null, the point estimates (on a log HR scale) for DTIC + IFN and non-DTIC are sufficiently large to defend the two-dimensional models. (Furthermore, ignoring the treatment effects in terms of shape, i.e. assuming a constant HR, implies that the uncertainty in model shape is not captured with the meta-analysis models.) Adding the treatment-by-covariate interaction term notably affected the treatment effect in terms of scale (*d*_0*Ak*_). The treatment-by-covariate interaction for non-DTIC vs. DTC (−0.02) was different than the interaction term obtained with a model with a constant interaction term (0.05), which implies that the assumption of a constant covariate interaction can be challenged.

**Table 2:** Model parameters for fixed and random effects two-dimensional (Weibull) network meta-analysis models with and without treatment-by-covariate interaction

	**Fixed effects model**		**Random effects model (model 1)**		Random effects model with treatment specific covariate interaction* (model 2)		Random effects model with constant treatment-by-covariate interaction* (model 3)	
	**Median of posterior distribution**	**95% Credible Interval**	**Median of posterior distribution**	**95% Credible Interval**	**Median of posterior distribution**	**95% Credible Interval**	**Median of posterior distribution**	**95% Credible Interval**
Pooled estimate for difference in scale *β*_*0*_								
DTIC + IFN vs. DTIC (*d*_*0AB*_)	−0.16	(−0.63; 0.33)	−0.22	(−0.76; 0.23)	−0.04	(−0.54; 0.47)	−0.18	(−0.58; 0.39)
DTIC + non-IFN vs. DTIC (*d*_*0AC*_)	−0.07	(−0.46; 0.34)	−0.19	(−0.65; 0.30)	−0.16	(−0.66; 0.32)	−0.10	(−0.51; 0.35)
non-DTIC vs. DTIC (*d*_*0AD*_)	−0.27	(−0.63; 0.13)	−0.30	(−0.88; 0.24)	−0.17	(−1.40; 1.11)	−0.43	(−1.12; 0.06)
Pooled estimate for difference in shape *β*_*1*_								
DTIC + IFN vs. DTIC (*d*_*1AB*_)	0.13	(−0.08; 0.33)	0.14	(−0.04; 0.34)	0.12	(−0.07; 0.30)	0.16	(−0.04; 0.30)
DTIC + non-IFN vs. DTIC (*d*_*1AC*_)	−0.06	(−0.23; 0.11)	−0.02	(−0.19; 0.15)	−0.04	(−0.21; 0.13)	−0.05	(−0.19; 0.10)
non-DTIC vs. DTIC (*d*_*1AD*_)	0.04	(−0.17; 0.23)	0.06	(−0.15; 0.27)	0.09	(−0.11; 0.29)	0.03	(−0.16; 0.21)
Estimate for covariate effect (*βx*)								
DTIC + IFN vs. DTIC (*βx*_*AB*_)					0.06	(−0.02; 0.16)	0.05	(−0.01; 0.12)
DTIC + non-IFN vs. DTIC (*βx*_*AC*_)					0.06	(−0.05; 0.18)	0.05	(−0.01; 0.12)
non-DTIC vs. DTIC (*βx*_*AD*_)					−0.02	(−0.21; 0.19)	0.05	(−0.01; 0.12)
Between study variance (heterogeneity) in scale			0.21	(0.02; 0.56)	0.19	(0.01; 0.52)	0.18	(0.01; 0.55)

* Covariate value set at the average across studies.

**Figure 4:**
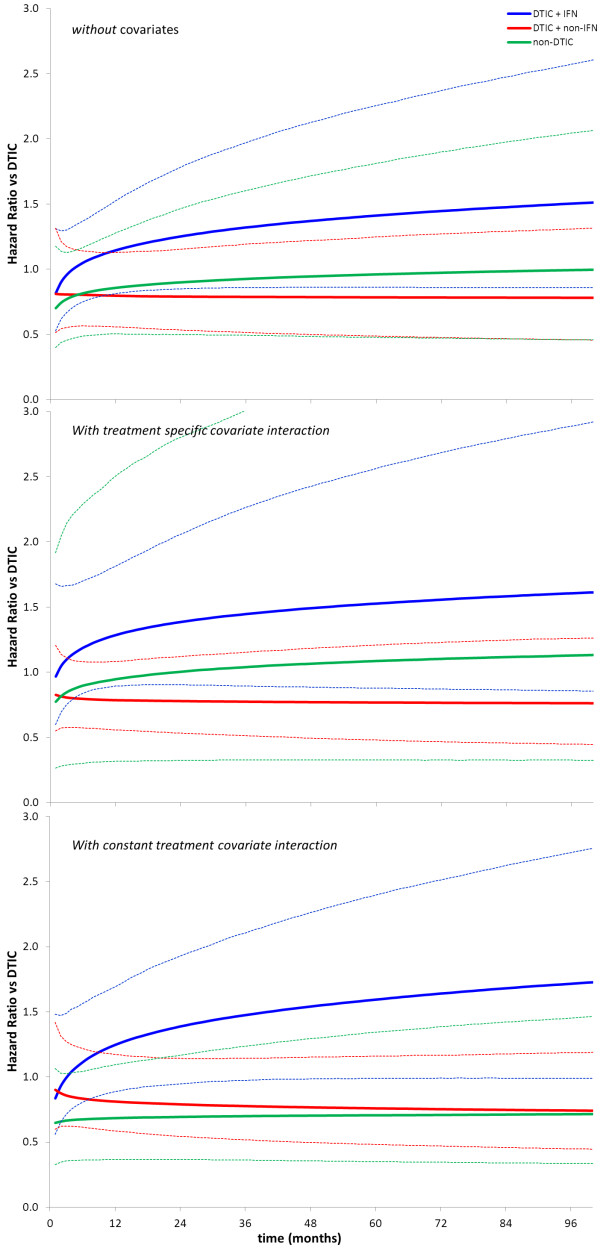
**Treatment effects of different interventions relative to DTIC as obtained with random effects Weibull network meta-analysis expressed as hazard ratio (and 95% credible interval).A**) Results without adjustment for differences in study date; **B**) Results after adjustment for differences in study date assuming treatment specific covariate interactions. Hazard ratios are presented corresponding to the average covariate value; **C**) Results after adjustment for differences in study date assuming constant covariate interaction. Hazard ratios are presented corresponding to the average covariate value

Based on the pooled treatment effects regarding the scale and shape of each intervention relative to DTIC (*d*_0*Ak*_, and *d*_1*Ak*_, with *k = B,C,D,* corresponding to respectively DTIC + IFN, DTIC + non-IFN and non-DTIC) the resultant HRs as a function of time were obtained according to ln(*HR*_*Ak*_) = *d*_0*Ak*_ + *d*_1*Ak*_ · ln(*t*). Figure 4A reflects the HRs over time (along with 95% credible intervals) with a random effects model without adjustment for differences in study date across studies and comparisons. Figure 4B shows the HR over time after adjustment for differences in study date using a random effect model with treatment specific covariate interactions (model 2). The HRs over time are presented for the average study date of all studies. Figure 4C shows the HRs over time after adjustment for study date using a random effect model with a constant treatment-covariate interaction (model 3). Comparing Figure 4A with Figure 4B and 4c illustrates the effect of ignoring the variation in study date across the different comparisons.

**Figure 5:**
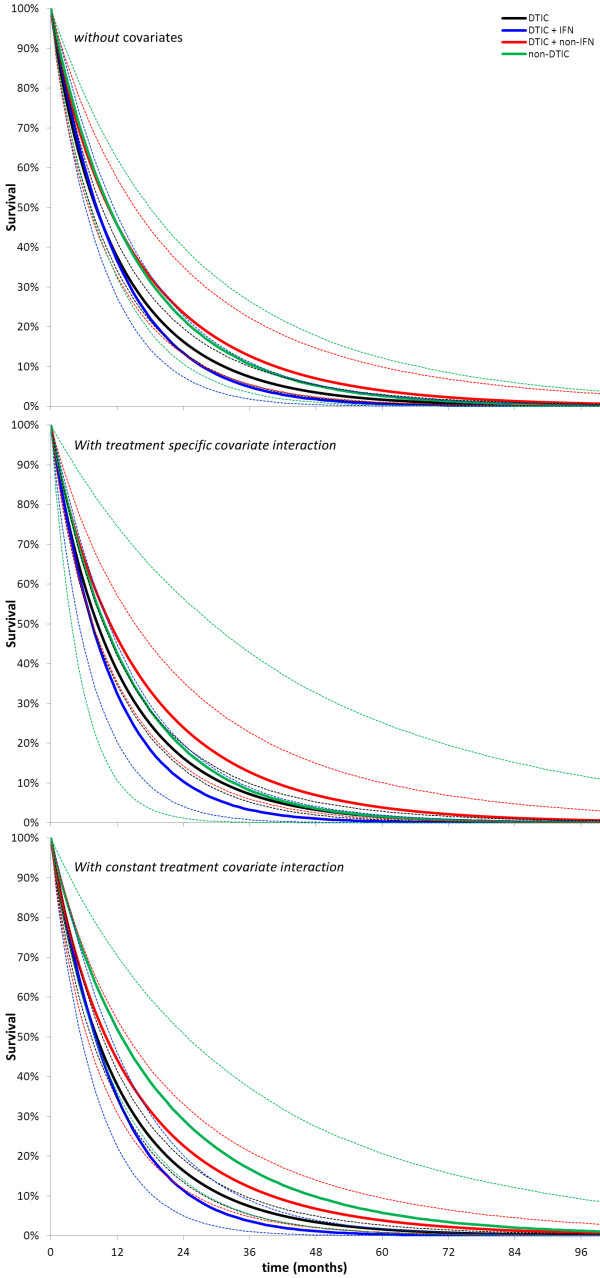
**Survival (and 95% credible interval) with different interventions as obtained with random effects Weibull network meta-analysis.A**) Results without adjustment for differences in study date; **B**) Results after adjustment for differences in study date assuming treatment specific covariate interaction. Results are presented corresponding to the average covariate value across studies; **C**) Results after adjustment for differences in study date assuming constant covariate interaction. Results are presented corresponding to the average covariate value

By applying the treatment effects on scale and shape as obtained with model 1, 2 and 3 (Table 2) to an average scale and shape of the studies with DTIC, an expected scale and shape was obtained for the other interventions. The corresponding survival functions for each of the four interventions are presented in Figure 5. This figure allows for comparisons of survival proportions at different time points (including median survival), as well as comparisons of expected (i.e. mean) survival, which is useful for cost-effectiveness evaluations. In Table 3 differences in expected survival are presented for the different random effects models.

**Table 3:** Difference in expected survival (in months) between interventions as obtained with random effects network meta-analysis model with and without treatment-by-covariate interaction

	**Random effects model without covariate interaction (model 1)**		**Random effects model with treatment specific covariate interaction (model 2)**		**Random effects model with constant treatment-by-covariate interaction (model 3)**	
	**Median of posterior distribution**	**95% Credible Interval**	**Median of posterior distribution**	**95% Credible Interval**	**Median of posterior distribution**	**95% Credible Interval**
DTIC + IFN vs. DTIC	−1.12	(−4.20; 3.49)	−2.46	(−5.72; 1.91)	−1.90	(−5.10; 2.22)
DTIC + non-IFN vs. DTIC	3.63	(−1.72; 10.75)	3.77	(−1.04; 10.72)	3.21	(−2.39; 9.65)
non-DTIC vs. DTIC	2.66	(−2.38; 13.49)	1.16	(−7.51; 25.76)	6.60	(−0.78; 21.39)
DTIC + non-IFN vs. DTIC + IFN	4.71	(−1.38; 11.37)	6.26	(0.26; 13.29)	5.13	(−1.15; 11.50)
non-DTIC vs. DTIC + IFN	3.81	(−2.40; 14.11)	3.46	(−6.00; 27.88)	8.48	(−0.65; 24.00)
non-DTIC vs. DTIC + non-IFN	−0.84	(−9.15; 10.3)	−2.79	(−13.90; 22.17)	3.27	(−6.72; 20.74)

**Figure 6:**
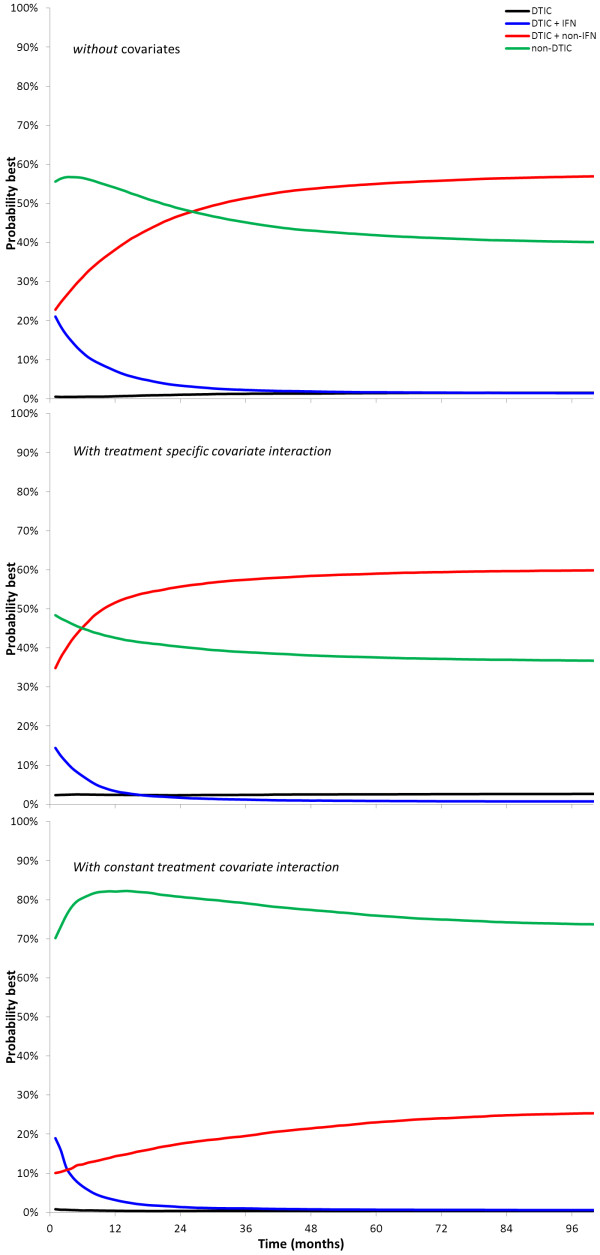
**Probability of greatest survival up to a certain time point (AUC) with different interventions as obtained with random effects Weibull network meta-analysis.A**) Results without adjustment for differences in study date; **B**) Results after adjustment for differences in study date assuming treatment specific covariate interaction. Results are presented corresponding to the average covariate value across studies; **C**) Results after adjustment for differences in study date assuming constant covariate interaction. Results are presented corresponding to the average covariate value

Given the Bayesian approach, the probability that a certain treatment shows the greatest survival at different time points was presented based on the posterior distribution of the estimated survival proportions over time (Figure 6).

**Figure 7:**
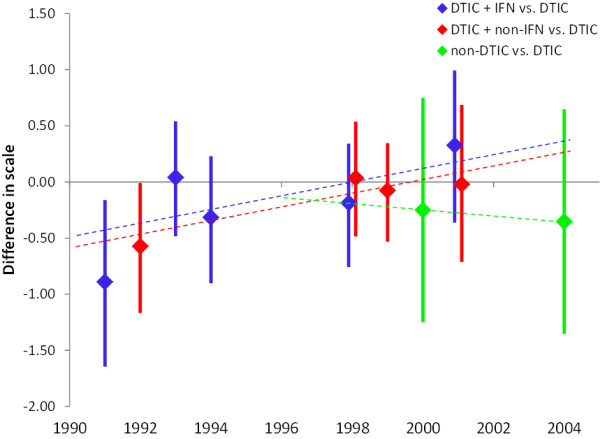
**Independent study specific differences in scale of DTIC + IFN, DTIC + non-IFN, and non-DTIC relative to DTIC as a function of publication year.** The associations between treatment effects in terms of scale and publication year as obtained with model 2 are presented with the dashed lines

To illustrate the relevance of the covariate publication date for this analysis, the study specific differences in scale of DTIC + IFN, DTIC + non-IFN, and non-DTIC relative to DTIC are presented as a function of publication year in Figure 7. (These study specific estimates were obtained with a model similar to Model 1, but now assuming independent study specific differences in scale, rather than exchangeable effects as obtained the random effects model.) From Figure 7 it can be inferred that for the comparisons DTIC + IFN versus DTIC and DTIC + non-IFN versus DTIC the treatment effects in terms of scale show an increasing trend over the years. Furthermore, the non-DTIC versus DTIC studies were performed at later point in time than the other studies. Given this imbalance, adjustment for publication date as a proxy for changing medical care is justified for this indirect comparison.

## Discussion

In this paper, the network meta-analysis models proposed by Ouwens et al. and Jansen for survival data are extended with treatment-by-covariate interactions to explain heterogeneity and possibly to adjust for confounding bias due to inconsistency [7,8]. The primary advantage of these evidence synthesis models for survival data is that they do not rely on a proportional hazards assumption across studies and comparisons, and any inconsistency due to imbalance in known and measured treatment effect modifiers can be (partly) adjusted for. Although models to incorporate covariates in network meta-analysis have been presented before, this is the first application where treatment effects act on two separate parameters [10-14].

Since the treatment effect acts on both scale and shape with these network meta-analysis models, differences in treatment effect modifiers across studies can (in principle) cause heterogeneity and inconsistency in terms of both the scale and shape. Accordingly, treatment-by-covariate interactions to explain heterogeneity or minimize inconsistency can be of a multidimensional nature as well. However, identifiability of models with treatment-by-covariate interactions for both scale and shape may be very challenging. Under the assumption that heterogeneity of treatment effects only act on the scale parameter, we need at least 2 studies for at least one of the treatment comparisons in the network to estimate a corresponding random effects model. If also covariate interactions are assumed for this effect, even more studies are needed. If we want to estimate heterogeneity and/or treatment-by-covariate interactions in terms of shape, we run in the additional challenge of decreasing sample size and event counts for longer follow-up time points, thereby making the estimation of any shape related parameter beyond a fixed treatment effect challenging. To ensure model identifiability, it is recommended to use network meta-analysis models with fixed treatment effects in terms of shape and only heterogeneity and treatment-by-covariate interactions regarding treatment effects in terms of the scale. Given the presence of a time related treatment effect in these models (i.e. shape), it is unlikely that study characteristics, patient characteristics, and contextual factors (which are most likely unaffected by time) have an impact on heterogeneity and inconsistency beyond the constant component of the treatment effect (i.e. the scale). More complex models with heterogeneity and covariate interactions in terms scale and shape parameters are only expected to be feasible if a large number of studies with sufficient number of events for longer follow-up times are available.

To estimate treatment specific covariate interaction terms (Model 2) we need sufficient covariate variation across the studies for each intervention *k* relative to an overall anchor treatment (A). For models with a constant covariate interaction term (Model 3) we have fewer parameters to estimate and these models are therefore easier to identify. Furthermore, for these models we do not necessarily need spread in the covariate across studies for each type of comparison relative to A; we only need sufficient variation across studies comparing any intervention relative to treatment A. An alternative approach to estimate treatment specific covariate interaction terms is a model with exchangeable interaction effects described by a normal distribution [10]. Such a model allows interaction estimates that shrink towards a common mean, thereby improving parameter estimation.

This network meta-analysis was based on survival proportions extracted from published Kaplan-Meier curves, used to calculate the incident number of deaths and patients at risk per interval according to an algorithm described by Jansen (See Additional file 1 of this paper) [7,8]. Guyot et al. recently proposed an improved algorithm to reconstruct the data from published Kaplan-Meier curves, which provides a closer approximation of the censoring times, thereby possibly improving the accuracy in terms of the number of patients at risk and allowing greater accuracy of the uncertainty in model parameter estimates [29].

In the present paper, covariate adjustment was based on study-level or aggregate level data. A challenge with meta-regression models using study level data is that the association between a patient characteristic and the treatment effect of the studied interventions at the study level may not reflect the individual level effect-modification of that covariate [30,31]. Hence, the models can only be used with study level data when there is between-study or between-comparison variation with limited variation in effect modifiers within studies. This is typically the case for study design characteristics or characteristics of the intervention such as dose. However, in the case of imbalances in baseline patient characteristics across comparisons, variation in these characteristics is often present within studies, in which case patient level data is required to ensure valid adjustment for inconsistency. Furthermore, patient-level data provides a greater opportunity to explore differences in effects among subgroups. However, obtaining patient-level data for all RCTs in the network may be considered unrealistic. If there is only individual patient data available for a subset of trials, combining individual patient data for this subset with study level data from the other studies provides a useful alternative [31].

The proposed models can also be used to adjust for differences in terms of baseline risk or placebo response across the trials. The placebo response reflects the impact of all study or patient characteristics that have an impact on the outcome; the placebo response summarizes the impact of prognostic factors (i.e. the study effect). If there is an association between the placebo response and treatment effects, it implies that some or all of the study and patient characteristics that are prognostic factors of the outcomes are also treatment effect modifiers. Adjustment for the placebo response partly mitigates inconsistency or bias due to an imbalance in effect modifiers across comparisons [9,10]. This may be important given the challenge of adjusting for multiple differences in effect modifiers using study-level data and the limited feasibility of accessing individual patient data for all trials. A limitation of adjusting for baseline risk in a network meta-analysis is the (theoretical) possibility of introducing collider stratification bias [9].

Although network meta-analysis where the treatment effects contain a time related aspect has obvious advantages in terms of model fit to the data, the presentation of the results might need some familiarization. The advantage of presenting HRs as a function of time (Figure 4) is that time varying treatment effects can be identified, but a possible disadvantage is the challenge to identify whether one treatment is overall favourable over another, especially when the HR curves cross. Pooled treatment specific survival curves as shown in Figure 5 provide information on commonly understood concepts like median survival and survival at different time points. A disadvantage, however is that a baseline survival curve with treatment A needs to be defined in order to translate the HR curves as obtained with the network meta-analysis into (pooled) survival curves by treatment. In the current example the pooled curve with treatment A was based on the average of study specific nuisance parameters for scale and shape of all treatment A (i.e. DTIC) controlled trials.

Within a Bayesian framework it is possible to calculate the probability of being the best treatment out of all those treatments assessed, or second best, third best, etc. [32]. With the time varying treatment effects obtained from the network meta-analysis models presented in this paper there are different options to create probabilistic summaries of treatment effects. We can either present a probabilistic summary based on a collapsed measure of the time varying treatment effects, or present probabilistic summaries as a function of time. Examples of the first category are based on the median survival or expected survival obtained from the pooled survival curves. Probabilistic summaries over time can be based on the HR at each time point, the survival proportion at each time point, or the area under the survival curve (AUC) to the left of each time point. Figure 6 is based on this concept and summarizes the cumulative treatment effect. Additional research is required to understand the sensitivity of these different probabilistic measures to the estimated underlying basic model parameters for the treatment effect, and provide some guidance.

A multidimensional network meta-analysis model for survival data is extremely useful for cost-effectiveness analysis because estimates of expected survival differences of competing interventions are less likely to be biased [7,8]. The extension of the two-dimensional model to include covariates allows for an evaluation of patient subgroups for which the clinical or cost effectiveness of the technology might be expected to differ from the overall population. Although the current analysis focused on overall survival, this method can be applied to any time to event outcomes. It may be of interest to extend the model to evaluate both progression-free and overall survival simultaneously using a multivariate approach, as has been proposed by Welton et al. [33]. This will avoid any inconsistency between clinical evidence synthesis and economic evaluations based on models with differences in quality of life before and after disease progression.

## Conclusion

Adding treatment-by-covariate interactions to multidimensional network meta-analysis models for published survival curves can be worthwhile to explain systematic differences across studies and to reduce inconsistencies. An additional advantage is that heterogeneity in survival data can be addressed. These models are not only useful for comparative effectiveness evaluation, but also provide an opportunity to ensure consistency within a cost-effectiveness analysis. In the Additional file 1 the data requirements to perform analysis with these kinds of models are outlined.

## Competing interests

The authors declare that they have no competing interests.

## Authors’ contributions

JJ and SC are both responsible for the development of the methods, analysis of the example and writing of the manuscript. Both authors read and approved the final manuscript.

## Pre-publication history

The pre-publication history for this paper can be accessed here:

http://www.biomedcentral.com/1471-2288/12/152/prepub

## Supplementary Material

Additional file 1 **Data requirements to perform network meta-analysis of published survival curves.** (DOC 64 kb) Click here for file
