# Supplementary material for: Meta-regression models to address heterogeneity and inconsistency in network meta-analysis of survival outcomes
Source: BMC Med Res Methodol. 2012 Oct 8;12:152. doi: 10.1186/1471-2288-12-152 (PMC3570315; doi:10.1186/1471-2288-12-152)
Supplement: Additional file 1 — Data requirements to perform network meta-analysis of published survival curves. (DOC 64 kb) [file 1471-2288-12-152-S1.docx]

**Data requirements to perform network meta-analysis of published survival curves**

- We need an evidence base that consists of randomized controlled trials, and each trial has at least one intervention in common with another; one network of randomized controlled trials.
- For each trial and intervention in the network, published Kaplan-Meier curves for the time-to-event outcome of interest are required, preferably with numbers of patients at risk over time. Differences in length in follow-up do not matter.
- It is necessary to digitally scan the complete reported Kaplan-Meier curve to obtain survival proportion over time. DigitizeIt (http://www.digitizeit.de/) software performs well. The Kaplan-Meier curves are read into the software by defining the axes and clicking on the select points from the Kaplan-Meier curve. In principle, every step of the Kaplan-Meier curves should be captured, i.e. scan each point where a vertical part of the Kaplan-Meier curve ‘changes’ into a horizontal part. It is important that these data points are captured accurately.
- Use an algorithm to create a data set that can be used for the analysis based on the scanned event proportions and reported at-risk population at different time points. Here we describe the approach used for the example analysis in the current paper which is based on the algorithm described in Jansen, 2011. (An alternative, more advanced approach to model censoring, has been recently published by Guyot et al. 2012) .The available follow-up time period for each arm of each trial is divided in *q* sequential short time intervals $\left[t_{1},t_{2}\right],\left(t_{2},t_{3}\right],...,\left(t_{q},t_{q+1}\right]$ with $t_{1}$=0. For each time interval *m* =1,2,3,…,*.q*. the conditional survival probability is calculated based on the scanned survival proportions according to $\frac{S\left(t_{m+1}\right)}{S\left(t_{m}\right)}$. If there is no survival proportion available for a specific time point, a corresponding estimate for $S\left(t_{m}\right)$is obtained by linear interpolation of the first available extracted scanned survival proportions before and after this time point. Given the reported numbers at risk $n_{m}$at the beginning of the m^th^ interval and the event probability for that interval, the actual number of events $r_{m}$for each interval will be calculated. As such, it is desirable to have the time intervals defined in such a way that (some) time points $t_{m}$ are aligned with the time point for which the size of the at-risk population is reported below the published Kaplan-Meier curve. The population at risk at the beginning of each interval $n_{m}$is extracted from the reported number below the Kaplan-Meier curve, or imputed based on the reported size of the at-risk population at subsequent time points according to $n_{m}=n_{m+1}/\frac{S\left(t_{m+1}\right)}{S\left(t_{m}\right)}$. With this approach we implicitly assume that censoring occurs before the events happen within a time interval. If there is no information on the at risk population for time intervals beyond the at risk population reported at a certain time point, the assumed at risk population can be calculated according to $n_{m+1}=n_{m}-n_{m}\left(\frac{S\left(t_{m}\right)-S\left(t_{m+1}\right)}{S\left(t_{m}\right)}\right)$. The disadvantage is that censoring is ignored. For each interval the number of events is calculated according to $r_{m}=n_{m}\left(\frac{S\left(t_{m}\right)-S\left(t_{m+1}\right)}{S\left(t_{m}\right)}\right)$.
- The time intervals do not have to have the same length. In general, the shorter the intervals the more variation in the hazard function will be picked up, but if the intervals are too short the number of events will be zero for many subsequent intervals. Hence, choose the interval length as a compromise to meet these requirements.
- Create a WinBUGS dataset as presented here below.
- To incorporate study-level covariates in the models to explain heterogeneity and adjust for inconsistency, we need the value of this covariate for each study. (If the study level covariate is missing, a value might be imputed based on other study and patient characteristics and information from other studies, but it is recommended to capture uncertainty in this imputed value as well.)

**WinBUGS code for random effects network meta-analysis model with a two-dimensional treatment effect and (constant) treatment-by-covariate interactions**

Model{

**#Transforming time depending on the survival function**

for (i in 1:N){

# For Weibull P1=0, for Gompertz P1=1

timen1[i]<-(equals(P1,0)*log(time[i]) + (1-equals(P1,0))*pow(time[i],P1) ) #transformation of time in months

**# likelihood**

r[i]~dbin(p[i], n[i])

p[i]<-1-exp(-h[i]*dt[i]) # cumulative hazard over interval [t,t+dt] expressed as events per person-month

**#Random effects model**

log(h[i])<- Beta[s[i],a[i],1]+Beta[s[i],a[i],2]*timen1[i]

}

for (l in 1:Ns){

w[l,1]<-0

delta[l,1]<-0

for (ll in 1:na[l]){

Beta[l,ll,1]<-mu[l,1]+delta[l,ll]

Beta[l,ll,2]<-mu[l,2]+d[t[l,ll],2]-d[t[l,1],2]

}

for (ll in 2:na[l]){

delta[l,ll]~dnorm(md[l,ll],taud[l,ll])

md[l,ll]<-d[t[l,ll],1]-d[t[l,1],1] +sw[l,ll]+sum(betax[l,ll,])

w[l,ll] <- (delta[l,ll] - d[t[l,ll],1] + d[t[l,1],1])

sw[l,ll] <- sum(w[l,1:ll-1])/(ll-1)

taud[l,ll] <- tau *2*(ll-1)/ll

# Covariate effects

for (ii in 1:nc) {

betax[l,ll,ii]<-(beta_x[t[l,ll],ii]-beta_x[t[l,1],ii]) *(x[l,ii]-sum(x[,ii])/Ns)

}

}

}

**#Priors**

# Priors for study effects

for (j in 1:Ns){

mu[j,1:2] ~ dmnorm(mean[1:2],prec2[,]) }

# Treatment effect is zero for reference treatment

d[1,1]<-0

d[1,2]<-0

#Covariate effect is zero for reference treatment

for (ii in 1:nc) {

beta_x[1,ii]<-0 }

# Priors for treatment effect

for (k in 2:Ntx){

d[k,1:2] ~ dmnorm(mean[1:2],prec2[,])

# Priors for treatment specific covariate effects

for (ii in 1:nc) {

beta_x[k,ii]~ dnorm(0,.0001)

}

}

# Alternative: Prior for constant covariate effects

#for (ii in 1:nc) {

#beta_x[k,ii]<-Beta_x[ii]

#}

#}

#for (ii in 1:nc) {

#Beta_x[ii]~ dnorm(0,.0001)

#} #end alternative prior

# Prior for between study heterogeneity

sd~dunif(0,2)

tau<-1/(sd*sd)

**#Output**

for (m in 1:maxt){

time1[m]<-(equals(P1,0)*log(m) + (1-equals(P1,0))*pow(m,P1) )

}

# Hazard Ratios over time for centered covariate values

for (ntx in 1:Ntx-1){

for (nntx in ntx+1:Ntx){

for (m in 1:maxt){

log(HR[ntx,nntx,m])<-(d[nntx,1]-d[ntx,1])+(d[nntx,2]-d[ntx,2])*time1[m]

}

}

}

} # Program ends

**DATA**

list(P1=0,

N=478, Ns=10, Ntx=4,

maxt=100, nc=1,

mean=c(0,0),

prec2 = structure(.Data = c(

0.0001,0,

0,0.0001

), .Dim = c(2,2))

)

# Treatment comparison by trial. Each row represents one study. t[,1] is the comparator treatment in arm 1, t[,2] is the intervention treatment in arm 2, etc. na[] is the number of arms per trial

t[,1] t[,2] t[,3] na[]

1 2 NA 2

1 2 NA 2

1 2 NA 2

1 2 NA 2

1 2 3 3

1 3 NA 2

1 3 NA 2

1 3 NA 2

1 4 NA 2

1 4 NA 2

END

# Study level covariate value.

x[,1]

1994 #Bajetta

1993 #Thomson

2001 #Young

1991 #Falkson 91

1998 #Falkson 98

1992 #Cocconi

2001 #Chiarion

1999 #Chapman

2004 #Avril

2000 #Middleton

END

# Events per interval. Each row represents one time interval. s[] is study identifier, r[] is number of events in interval, n[] is patients at risk for event in interval, a[] is arm in trial, time[] is cumulative time at end of interval, dt[] is the length of interval

s[] r[] n[] a[] time[] dt[]

1 4 82 1 2 2

1 9 69 1 4 2

1 12 60 1 6 2

1 6 48 1 8 2

1 3 32 1 10 2

. . . . . .

. . . . . .

. . . . . .

1 . . 2 . .

1 . . 2 . .

1 . . 2 . .

. . . . . .

. . . . . .

. . . . . .

2 . . 1 . .

2 . . 1 . .

2 . . 1 . .

. . . . . .

. . . . . .
